# Dicarbonyl L-Xylulose Reductase (DCXR), a “Moonlighting Protein” in the Bovine Epididymis

**DOI:** 10.1371/journal.pone.0120869

**Published:** 2015-03-27

**Authors:** Ayodélé Akintayo, Christine Légaré, Robert Sullivan

**Affiliations:** Centre de Recherche du Centre Hospitalier Universitaire (CHU) de Québec, Département d’Obstétrique, Gynécologie et Reproduction, Université Laval, Faculté de Medicine, Québec, Canada; Clermont Université, FRANCE

## Abstract

During maturation and the acquisition of their fertilization potential, male germ cells are subjected to various sequential modifications that occur in the epididymis. Protein addition, reorganization or withdrawal, comprise some of these modifications. Dicarbonyl L-xylulose reductase (DCXR), a multifunctional protein involved in various enzymatic and protein interaction processes in different physiological systems, is one of the proteins added to spermatozoa in the epididymis. DCXR is a well-conserved protein with multiple characteristics including enzymatic activities and mediation of cell-cell interaction. In this study, we characterized the *DCXR* gene and protein expression in the bovine epididymis. Dicarbonyl L-xylulose reductase mRNA is differentially expressed in the caput, corpus, and cauda epididymide epithelial cells with a higher level observed in the cauda region. Tissue protein expression follows the same pattern as the corresponding mRNA expression with a cytoplasmic and apical distribution in the corpus and cauda epithelial cells, respectively. The protein can also be found with a nuclear localization in cauda epididymidis epithelial cells. Dicarbonyl L-xylulose reductase is secreted in the epididymis luminal compartment in the soluble fraction and is associated with microvesicular elements named epididymosomes. In spermatozoa, the DCXR protein was found in the cytoplasmic and membranous fractions. Expression of the DCXR protein is higher on caput spermatozoa but finally shows a weak detection in semen. These data describe *DCXR* in the bovine epididymis and reveal that its behavior differs from that found in humans. It seems that, in this model, the DCXR protein might have a questionable involvement in the fertilization process.

## Introduction

Through physiological, biochemical, and molecular studies, it has been shown that the epididymis displays an extraordinary complexity at both the structural and functional levels [1]. This organ can be divided into three main regions based on their anatomical properties: the caput, the corpus, and the cauda followed by the more distal deferent duct. Each of these regions possesses a distinct gene expression pattern that relies on specific timing and fine regulation of epididymal gene profiles as documented by several microarray-based studies [2, 3]. Thus, the composition of this milieu varies along the different segments of the epididymis. This maturation organ provides spermatozoa with the required combination of various parameters in the specific microenvironment that is necessary for acquisition of their motility and fertilizing ability [4]. Numerous proteins secreted in the epididymal intraluminal compartment have been shown to interact with the maturing spermatozoa.

Dicarbonyl L-xylulose reductase (DCXR) being one of them, this member of the short-chain dehydrogenase/reductase superfamily is well-conserved among species. It is considered as a moonlighting protein as it is involved in different processes in the organism [5, 6]. Among these are: the regulation of the osmotic state of the cellular environment [7]; the detoxification of the cellular environment [8]; cell to cell interaction in the skin [9], and as previously identified by our group, its involvement in human spermatozoa/oocyte interaction; its presence correlating with men fertility [10]. DCXR expression in the epididymis has been described in other species such as mouse, rhesus monkey and hamster.

In this study, the bovine model was used to understand the role of the moonlighting protein DCXR in epididymal functions. Bull epididymides can be easily obtained from the slaughterhouse, allowing preparation of uncontaminated material from different subcompartement of epididymal segments such as spermatozoa suspension and soluble and particulate (epididymosomes) fraction from epididymal fluid. To our knowledge, this is the first evidence of DCXR protein in the bovine. The role of this epididymal protein in the sperm maturation process is discussed.

## Results

### Sequence analysis of bovine DCXR

**Fig 1:**
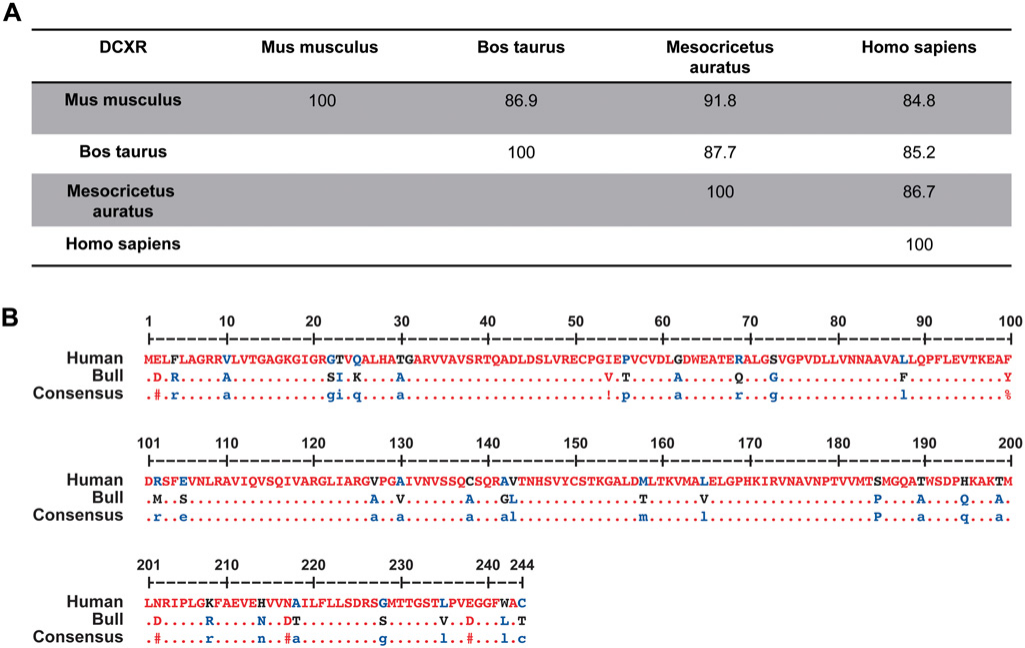
Mammalian DCXR sequence homology. (A) Homology among four different mammalian DCXR protein sequences (percentage). UniProtKB identification number: *Homo sapiens*: Q7Z4W1, *Bos taurus*: Q1JP75, *Mus musculus*: Q91X52, *Mesocricetus auratus*: Q91XV4. (B) DCXR protein sequence alignment between human and bull. Only sequence differences are indicated. The alignment was made with http://multalin.toulouse.inra.fr/multalin/ online software.

*In silico* sequence alignment of human, murine (*Mus musculus*), cricetine (*Mesocricetus auratus*), and bovine (*Bos taurus*) DCXR protein sequences reveals a high sequence homology, up to 91.8% between mouse and hamster (Fig. 1A). Human DCXR shares 85.2% homology with *Bos taurus* DCXR (Fig. 1A and B).

### DCXR mRNA distribution along the bovine epididymis

**Fig 2:**
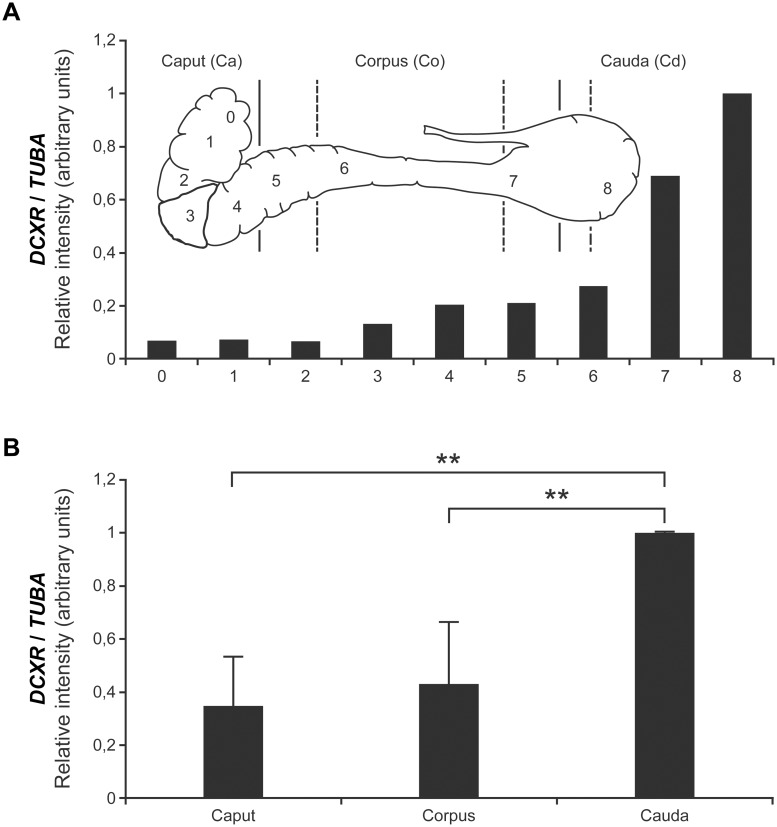
*DCXR* mRNA expression along the epididymis. (A) QPCR quantification on nine (9) different bovine epididymal sections from the caput to cauda (0 to 8). Inner plate is a schematic representation of the different bovine epididymal segments analyzed. (B) QPCR quantification on the three major sections (caput, corpus, and cauda) of the epididymis. Analysis made on five (5) different bovine epididymides. *DCXR* expression levels are normalized as a ratio of alpha-tubulin mRNA levels. ** = Significant difference by student’s t-test, t < 0.006.

**Fig 3:**
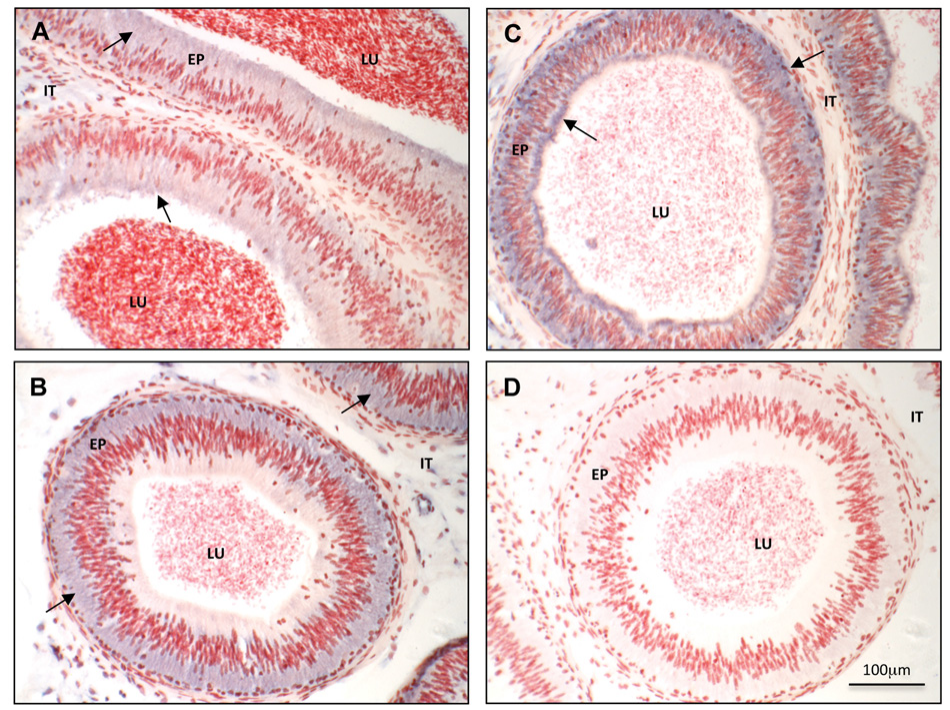
*In situ* hybridization localization of bovine *DCXR* mRNA on tissue cryosections of bovine caput (A), corpus (B, D), and cauda (C) epididymidis. Arrows indicate *DCXR* mRNA staining in blue with the DIG-labeled antisense probe (A, B, C) detected using an anti-DIG antibody coupled to alkaline phosphatase followed by incubation with NBT-BCIP substrate. D panel: corpus section probed with the negative control sense cRNA probes. Counterstaining with neutral red. LU = lumen; EP = epithelium; IT = interstitial tissue.

Nucleic acid sequence amplified from cDNA extracted from epididymal tissues shows 100% homology to bovine *DCXR* sequences registered in the GenBank database. Expression of the *DCXR* transcript increases along the epididymis (from caput to the cauda region), especially in the distal portions (Fig. 2A). Statistical analysis by Student’s t-test of QPCR data obtained from five different bulls shows a weak and non-significant difference between the caput and the corpus region but a significant difference between the cauda expression level and the two preceding regions (Fig. 2B). *In situ* hybridization was used to localize the *DCXR* gene expression pattern at the histological level. In agreement with the PCR results, antisense *DCXR* cRNA hybridization increases from the caput to the cauda epididymidis, with expression being in the majority, located to the epithelial cells. Some faint staining can be found in some blood vessels endothelial cells (S1 Fig.). The mRNA is uniformly located in the cytoplasm of caput epithelial cells (Fig. 3A). A more basal localization is observed in the corpus tissue, and a mixed basal and apical localization characterizes the cauda segment (Fig. 3B–C).

### DCXR protein levels along the bovine epididymis

**Fig 4:**
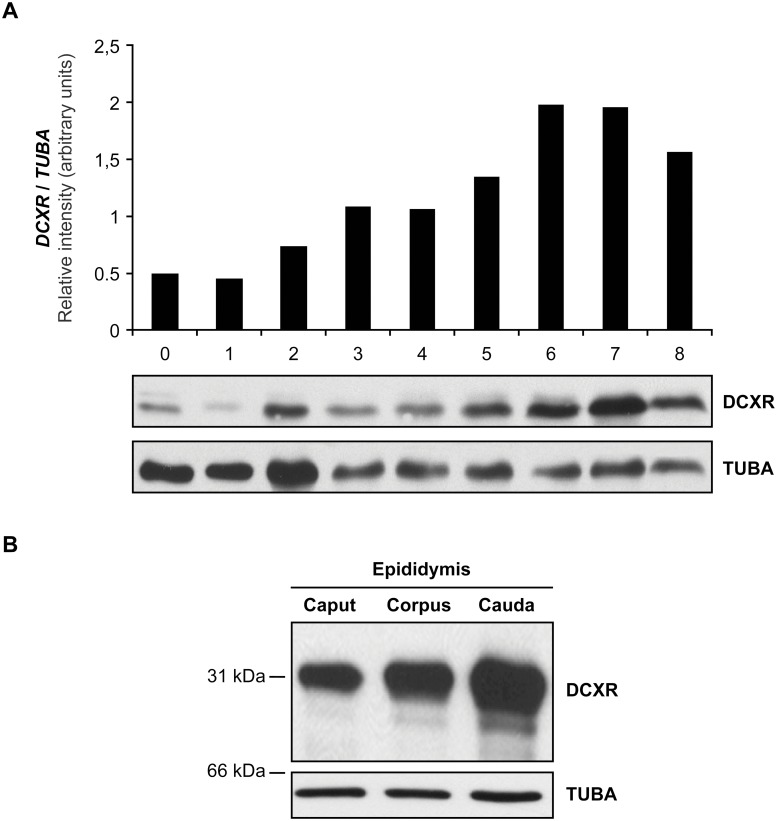
DCXR protein levels along the bovine epididymis. (A) Western-blot analysis on SDS protein extracts from nine (9) bovine epididymis segments, as illustrated in Fig. 2A, 20 μg per lane. (B) Western-blot analysis of the three major sections caput, corpus, and cauda epididymidis probed with anti-bovine recombinant DCXR antiserum. Results are representative of five independent experiments. An anti-tubulin antiserum was used as an internal loading control.

**Fig 5:**
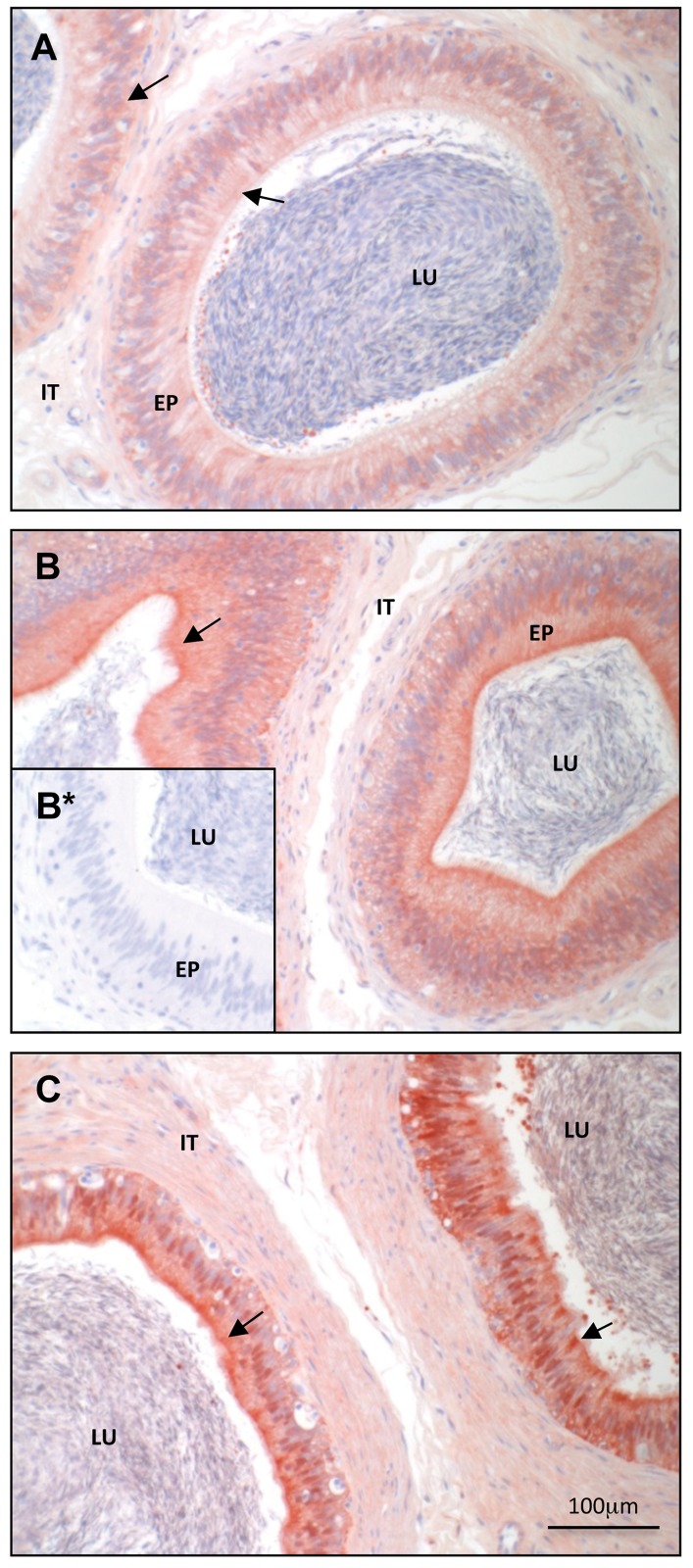
Immunohistochemical localization of bovine DCXR protein along the bovine epididymis. Caput (A), corpus (B and B*) and cauda (C) epididymidis using anti-DCXR antiserum (A, B and C). A pre-immune rabbit serum was used as a negative control (B*). Arrows indicate DCXR detected as a brown-red staining. The sections were counterstained in blue with Harris hematoxylin. LU = lumen; EP = epithelium; IT = interstitial tissue.

To characterize the expression pattern of the DCXR protein in the epididymis, the complete *DCXR* mRNA transcript from the cauda section was used to produce a his-tag conjugated recombinant full DCXR protein in a prokaryotic system, and the purified protein (S2 Fig.) was used to produce specific rabbit antisera against bovine DCXR. The specificity and efficiency of the antisera is shown in S3 Fig. and S4 Fig. Western blot analysis on protein extracts from caput, corpus, and cauda regions of the epididymis, revealed a protein of 32 kDa with the same expression pattern as the *DCXR* gene, *i*.*e*., increasing expression from the caput extract to the cauda (Fig. 4A and B). Immunohistochemical assay revealed that the DCXR protein is located in the epithelial cells of the epididymis and at a weaker level in some blood vessels (data not shown). The protein follows the same distribution pattern as the mRNA expression along the epididymis: low level and diffuse cytoplasmic localization in the caput (Fig. 5A), a strong cytoplasmic and apical expression in the corpus (Fig. 5B), and finally, a high level of expression in the basal and apical subcellular compartment in the cauda epididymal epithelium (Fig. 5C). One can notice a high staining in the apical cells of the caput epididymidis epithelium. This is not observed in the corpus or cauda epididymidis (S5 Fig. and S6 Fig.). The anti-DCXR anti-serum used is highly specific as shown by the absence of staining when a pre-immune serum (Fig. 5B inset) or the anti-DCXR sera pre-absorbed with the recombinant protein was used (data not shown).

### DCXR detection on spermatozoa

**Fig 6:**
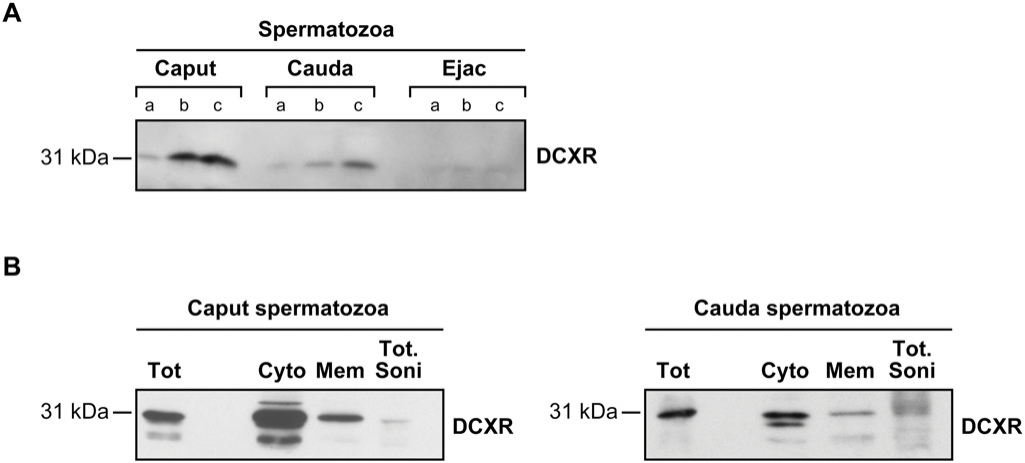
DCXR protein levels associated with spermatozoa. (A) Western blot analysis on SDS protein extract from an increasing amount of caput, cauda, and ejaculated spermatozoa pellets (a = 5 × 10^6^ b = 10 × 10^6^ c = 20 × 10^6^). (B) DCXR detection on total caput and cauda spermatozoa (Tot) and on subcellular fractions: 20 μg of cytoplasmic (cyto); membrane (mem) and sonicated remaining spermatozoa (Tot soni). Molecular weight is indicated on left (31 kDa).

Proteins extracted from washed spermatozoa from the caput; cauda epididymidis and semen were used for western blot assays. The amount of DCXR detectable on a constant number of caput spermatozoa varied among individuals (data not shown). The protein level decreased from the caput to the ejaculated spermatozoa where it was detected at low levels (Fig. 6A). The total amount of DCXR on the ejaculated spermatozoa also varied among individual bulls (S7 Fig.). Subcellular fractionation of caput and cauda epididymal spermatozoa revealed that DCXR is located in the cytoplasmic and membrane fraction with higher levels in sperm cytoplasm (Fig. 6B).

### DCXR in the epididymis luminal compartment

**Fig 7:**
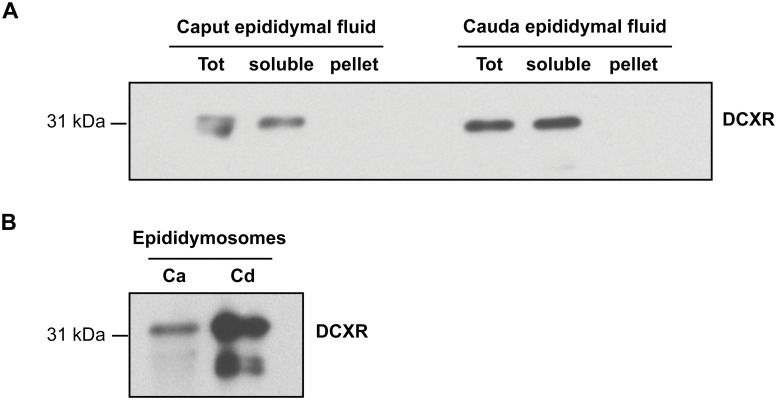
DCXR protein levels in the epididymal fluid. (A) Western blot analysis of DCXR protein expression in the caput and cauda epididymal fluid. Tot = total fluid 10 μg; soluble = equivalent volume of ultracentrifuged fluid; pellet = equivalent amount of epididymosomes. (B) DCXR detection in 10 μg of SDS extracts of epididymosomes from caput (Ca) and cauda (Cd) epididymidis. Detected with a rabbit anti-DCXR antiserum). Molecular weight is indicated on left (31 kDa).

The epididymis intraluminal fluid was analyzed by western blot assay for detection of DCXR protein. Total fluid from the distal regions of caput and cauda were fractionated into a soluble fraction and a membranous fraction (epididymosomes). Fig. 7 shows immunodetection of DCXR in a given amount of total fluid protein and its equivalent amount of soluble fraction, and epididymosomes. The DCXR protein is present in the fluid and is more abundant in the soluble fraction than in epididymosomes. However, the level of DCXR is still discernable in the caput and cauda epididymosomes with a higher signal in the distal segment (Fig. 7B).

### DCXR distribution in fluid epididymosomes

**Fig 8:**
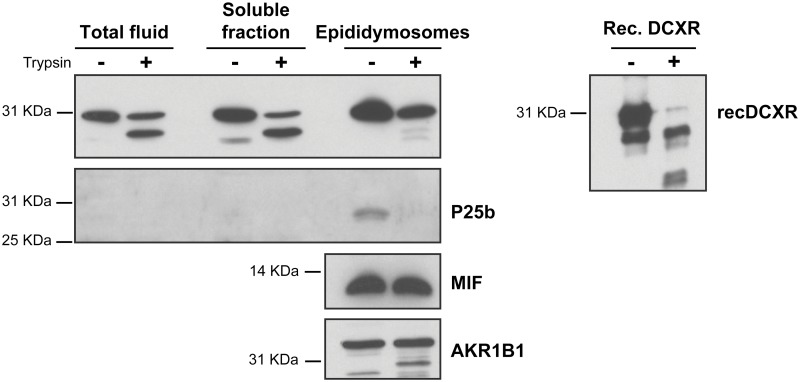
Trypsin digestion of cauda epididymidal fluid before ultracentrifugation (Total); after ultracentrifugation (soluble fraction); epididymosomes, and bovine recombinant DCXR protein. Western blot detection of DCXR (32 kDa), MIF (12 kDa), AKR1B1 (35 kDa) or P25b (28 kDa) protein from fluid; epididymosomes or recombinant bovine-DCXR (recDCXR) protein treated (+) or not (−) with trypsin (25 μg/ml). Proteins were detected with a rabbit anti-DCXR antiserum, a rabbit anti-P26h/P25b antiserum [11] for P25b, a rabbit anti-MIF antiserum [12], and a rabbit anti-aldose reductase (AKR1B1) antiserum [12]. The results are representative of three independent experiments. Molecular weight standards (kDa) are indicated on left.

It has been shown that epididymosomes are structures with compartmentalized proteins. In order to determine whether DCXR is compartmentalized in epididymosomes, the total caudal fluid, the soluble fraction, and the purified vesicles were exposed to proteolytic trypsin digestion. As shown in Fig. 8, immunoblot analysis indicated that DCXR is almost totally degraded in the total and soluble fraction, but only some of the protein is affected by proteolysis in the purified vesicles fraction. This suggests that the protein is found on both sides of the vesicles (internal and external). To confirm that the protein is insensitive to trypsin treatment as a result of its internal localization, the recombinant protein was treated with trypsin under the same conditions. The recombinant protein was affected by the treatment, confirming that DCXR is sensitive to protease cleavage in a soluble form, but not when associated with epididymosomes. As a control, trypsin-treated or untreated epididymosomes were immunoblotted to detect P25b, MIF and AKR1B1. As expected, P25b associated with epididymosomes was totally degraded by trypsin treatment. In contrast, MIF and AKR1B1 were unaffected by the protease treatment as a result of their internal localization in epididymosomes.

## Discussion

As a member of a well-conserved enzyme family, the DCXR protein shares high sequence homology among species [7], and shows 85.2% sequence homology between human and *Bos taurus* (Fig. 1A and B). As a moonlighting protein, DCXR has been shown to play different functions both in and out of cells depending on its subcellular environment [5]. The enzymatic activity of this protein is important for sugar metabolism [7] and cell detoxification [8, 13]. Furthermore, it appears to play a role in skin melanomas and prostatic adenocarcinomas [9, 14]. Of additional interest is that this epididymal protein acquired by spermatozoa during transit through the epididymis, is important in the spermatozoa-oocyte interaction in humans [10, 15].

The variation in *DCXR* gene expression along the bull epididymis was similar to, but not exactly the same as that observed in its human, hamster, and monkey counterparts [16–18]. In human epididymis, *DCXR* is highly expressed in the corpus region and decreases upon reaching the distal region [17]. In the bovine model, DCXR expression increases from the caput to the cauda of the epididymis. Using a specific in-house rabbit antibody raised against recDCXR, we showed that the encoded protein follows the same expression pattern as the corresponding mRNA and is localized in the epithelial cell cytoplasm of the caput and corpus tubules, but shows a predominantly apical localization in corpus (brush-border) and cauda epithelial cells. The protein is transferred from the epithelial cells to the luminal compartment of the epididymis by merocrine and apocrine secretion [1]. These secretory processes contribute to the increased amount of DCXR protein in the distal region fluid and in epididymosomes. In silico analysis on the bovine *DCXR* sequence returned no obvious signal peptide. Nevertheless DCXR is targeted to the membrane and secreted as a soluble protein in the fluid as well as being integrated into micro-vesicles. The sequence analysis also output three possible N-glycosylation and one N-acetylation (methionine) sites. These could explain the difference between the expected 26 kDa molecular weight and the 32 kDa observed in our results. By homology with other proteins associated with epididymosomes, the fact that the DCXR protein can be found on micro-vesicles may suggest transfer to the spermatozoa during their maturation; however, our results demonstrate a decrease in sperm-associated DCXR during epididymal transit. Thus, DCXR is not transferred, but is selectively removed during the sperm maturation process in bovine. As observed by Sudo and collaborators [19], western blot assays reveal a lower molecular weight DCXR band with relatively high signal in spermatozoa, cauda epididymosomes and fluid, suggesting that part of the removed protein might be degraded or that its removal from the spermatozoa involved a proteolytic cleavage of DCXR.

In human, the absence or reduced quantities of DCXR on ejaculated spermatozoa correlated with a decrease in fertility [20] thus, a role for the bovine counterpart of DCXR in the binding of the spermatozoa to the oocyte appears less likely given that the amount of DCXR on ejaculated spermatozoa is less abundant when compared with humans (Fig. 6A). Furthermore, addition of anti-DCXR antibodies or of purified recombinant DCXR to in vitro fertilization medium did not statistically interfere with sperm binding to bovine oocytes matured in vitro (S8A and B Fig.). As these results are quite different from those observed in humans [10, 17] this suggests that human and bovine DCXR do not have the same behavior. All together and as shown in Fig. 8, our data shows that this bovine DCXR behaves differently from P25b previously described by our laboratory [21]. In accordance with Ebert and collaborators [6], one can speculate that the antibody used in this study is able to distinguish DCXR from P25b, which seems to be the Cbr2 bovine homolog. Thus, in the bovine, Cbr2 seems to be more involved in the spermatozoa/oocyte interaction than DCXR.

A discrepancy in protein expression in the human epididymis compared with other species has already been documented [22]. However, the question concerning the role of this sperm maturation protein in the bull epididymis remains. As postulated by our laboratory [18] and others, the enzymatic role of DCXR in the kidney uronate cycle of glucose metabolism could be the same in the epididymis, even in the case of a low concentration of glucose (or any reducing hexose) in the epididymal fluid [23, 24]. The protein could also be involved in water absorption, or the killing of bacteria in the epididymal fluid by the production of xylitol, which is known to act as an osmolyte in the lung [25]. Water absorption in the epididymis is essential for sperm concentration, to maintain sperm viability during the storage period in the cauda epididymidis [26], and to facilitate interaction of the sperm with the different accessory gland secretions during ejaculation [27]. Furthermore, it has been postulated that sperm acquire osmolytes during their epididymal transit in preparation for the rapid change in osmotic environment upon ejaculation [28–31]. This could explain the presence of DCXR in the caput and corpus sperm. Another role for this moonlighting protein is the detoxification of cells and their environment by acting on carbonyl compounds. Indeed, high amounts of DCXR have been found in the cytoplasm of renal tubule cells involved in renal filtration [8, 13, 19]. It has been reported that the DCXR protein can be found in the nuclear compartment of cells with increased expression of the protein in melanomas [9, 14]. We observed DCXR in the nuclei of bovine cauda epididymidis epithelial cells (data not shown): as these cells are not in a carcinomatous environment, but do contain the protein in their nucleus, one can hypothesize that the presence of the protein in a carcinomatous environment would have a protective purpose. Moreover, the increasing amount of DCXR in the fluid and tubule epithelial cells could correlate with a plausible increase of dead spermatozoa when approaching the cauda storage area in the epididymis. Thus, DCXR could play a role in the detoxification of fluid and tissues to protect living spermatozoa from the products of the dead spermatozoa.

In conclusion, our results demonstrate that the role of DCXR in the bovine model is different to that observed in humans with regard to the global expression pattern in the epididymis tissue. The bovine protein is nevertheless highly expressed and shows a unique pattern of expression in the male tract. As a moonlighting protein, bovine DCXR could have a role related to its enzymatic activity or binding properties that can certainly also be found in the human epididymis. Further investigation is warranted to determine the importance of this protein in the bovine spermatozoa maturation process.

## Materials and Methods

### Tissues and spermatozoa

Epididymidis tissues from sexually mature bulls were obtained from a commercial slaughterhouse (Abattoir Bolduc, 1997 Chemin des Érables, Buckland, QC) and stored on ice until arrival at the laboratory. The tissues was dissected and snap frozen for extraction of RNA or protein. The epididymal spermatozoa and fluid were recovered from caput tubules by applying a gentle pressure to the proximal region of the caput epididymis and by retrograde flushing in the cauda region, as previously described [32, 33]. Spermatozoa from the caput; cauda region and semen were washed before processing as described by Caballero and collaborators [34].

### RNA extraction and bovine DCXR cloning

Frozen tissues from caput, corpus, and cauda epididymidis were weighed, ground to a powder in liquid nitrogen, mixed with RNA extraction media (QIAGEN RNeasy mini kit) then snap frozen. Ribonucleic acids were extracted with TRIZOL (Invitrogen, Carlsbad, CA) and purified according to the manufacturer’s protocols. The RNA was dissolved in sterile DEPC-treated water and reverse-transcribed using the enzyme Superscript II (Invitrogen). Full-length *DCXR* cDNA was amplified from cauda epididymidis using specially designed forward and reverse primers (Table 1). The reaction conditions for standard PCR amplification were as follows: initial denaturation/enzyme activation at 95°C for 5 min followed by 30 cycles of denaturation at 95°C for 1 min, annealing at 57°C for 1 min, and extension at 72°C for 1 min. The amplified PCR product was purified using the QIAquick purification kit and cloned into pGEM-T. Sequencing was performed by the CHUQ Research Center Core Facility (CHUQ Research Center, Quebec, QC, Canada). The DNA fragment was excised with Nhe 1 and Xho 1 restriction endonucleases and introduced into pET21b to produce the recombinant protein (recDCXR).

**Table 1:** Polymerase chain reaction primers sequences used for this study.

	Forward	Reverse	Product length
**DCXR full length**	5’ CCA GCT AGC ATG GAT TTG AGA CTC GCG 3’	5’ CTA CTC GAG GGT AGC CAG GAA GCC 3’	735 bp
**DCXR shortened (QPCR)**	5’ AGT GAT GGC TGT GGA GCT TG 3’	5’ ATG GTG TCC ACC ACG TTC TC 3’	174 bp
**GAPDH (QPCR)**	5’ CCA CTC CCA ACG TGT CTG TT 3’	5’ TGA AGT CGC AGG AGA CAA CC 3’	153 bp
**TUBULIN A (QPCR)**	5’ TCC ATC CAC GTT GGC-CAG GCT 3’	5’ AGC CCC TGT CTC ACT GAA GAA 3’	159 bp

The underlined sequence contains the restriction enzyme sites added for cloning purposes. Primers were designed using NCBI Primer-BLAST online tool (http://www.ncbi.nlm.nih.gov/tools/primer-blast).

### Quantitative real-time PCR assay

Total RNA extracts from the different epididymis segments were processed for reverse transcription (as described above) and the *DCXR* fragment was amplified by quantitative polymerase chain reaction (QPCR). The reaction conditions were as follows for quantification by real-time PCR: initial denaturation/enzyme activation step (5 min at 95°C), 40 PCR cycles were performed as followed: 5 seconds, denaturation 95°C; 5 seconds, annealing (56°C); elongation at 72°C (20 seconds). Temperature of fluorescence acquisition was 68°C. Expression data were normalized to two housekeeping genes (glyceraldehyde-3-phosphate dehydrogenase (*GAPDH*), tubulin alpha (*TUB A*)) based on the approach proposed by Vandesompele *et al*. [35, 36]. Refer to Table 1 for primer sequences.

### *In situ* hybridization

The *in situ* hybridization assay was performed on epididymal tissues using digoxigenin (DIG)-labeled (Boehringer Mannheim, Laval, Canada) cRNA probes as previously described by Legare and collaborators [17]. In brief, the 8 μm epididymis cryosections were fixed in freshly prepared 4% paraformaldehyde in PBS for 5 min at room temperature, incubated for 10 min in 95% ethanol/5% acetic acid at −20°C, and rehydrated by successive passage through decreasing concentrations of ethanol diluted with DEPC-treated H_2_O. After unmasking the target RNA by enzymatic digestion, the tissues were prehybridized at 42°C for 3 h with preheated 250 μg/ml salmon sperm DNA in hybridization solution (0.3 M NaCl, 0.01 M Tris-HCl, pH 7.5, 1 mM EDTA, 1 × Denhardt’s solution, 5% dextran sulfate, 0.02% SDS, and 50% formamide). Sections were then incubated overnight at 42°C with 25 μl of 10 μg/ml heat-denaturated antisense or sense DIG-labeled cRNA according to the supplier’s instructions (Roche Diagnostics, Laval, Canada). After washing, nonspecific staining was blocked by pre-incubation for 1 h with 5% (vol/vol) heat-inactivated sheep serum in 0.2 M Tris-HCl, 0.2 M NaCl, and 3% Triton X-100. Hybridization reactions were detected by immunostaining for 2 h at room temperature with alkaline phosphatase-conjugated anti-DIG antibodies, diluted 1:1,000 in blocking solution. The hybridization signal was visualized after a 15- to 20-min incubation period with the phosphatase substrate nitroblue tetrazolium chloride and 5-bromo-4-chloro-3-indolylphosphate p-toluidine salt (Roche Diagnostics, Laval, Canada). Levamisole (2 mM; Sigma-Aldrich Chemical Co., Oakville, CA) was added to the reaction mixture to inhibit endogenous alkaline phosphatase. The reaction was stopped by immersing the slides in 1 mM EDTA, 0.001 M Tris-HCl, pH 7.5, followed by a 5-min wash in H_2_O. The tissues were counterstained with neutral red, dehydrated by passage through increasing concentrations of ethanol, cleared in xylene, and mounted with Permount (Fisher Scientific, Nepeau, Ontario, Canada). All sections were processed in parallel to allow comparison.

### DCXR antibody production

The *Escherichia coli* BL21 strain was transformed with the *DCXR* cDNA sequence cloned into the pEt-21b prokaryotic expression plasmid, and overexpression was induced with 0.5 mM isopropyl-β-D-thiogalactopyranoside (IPTG) for 3 h at 37°C. The protein was recovered from bacterial lysate and purified on ProBond resin (Invitrogen Life Technologies Inc., Burlington, Canada). Purification of the full-length recDCXR was evaluated by SDS-PAGE. Three hundred micrograms of recDCXR solubilized in PBS pH 7.4 was mixed with one volume of Freund’s complete adjuvant and injected into New Zealand rabbits. Three boosts of 150 mg of recDCXR emulsified in incomplete Freund’s adjuvant were injected monthly. This study was approved by the animal care committee of our institution, CPA-CHUQ (Comité de Protection des Animaux du centre Hospitalier Universitaire de Québec) (Permit Number: 201188–3) and in accordance with the CCACs (Canadian Council on Animal Care) recommendations. This rabbit polyclonal antiserum against recDCXR was used at 1/10,000 (vol/vol) for western blot and 1/2,000 (vol/vol) for immunohistochemistry.

### Other antibodies used in this study

Anti-tubulin alpha mouse monoclonal antibody (Sigma-Aldrich, Saint Louis, MO, USA) was used at 1/10,000 (vol/vol) for western blot assays. Anti-P26h/P25b rabbit polyclonal antibody was used at 1/10,000 (vol/vol) [11]. Anti-MIF rabbit polyclonal antibody was used at 1/3,000 (vol/vol) [12]. Anti-AKR1B1 (Aldose reductase) rabbit polyclonal antibody was used at 1/5,000 (vol/vol) [12]. Secondary antibodies for western blot experiments: goat anti-rabbit (GAR); goat anti-mouse (GAM) and rabbit anti-goat (RAG) IgG conjugated to horseradish peroxidase (Jackson Immunoresearch West Grove, PA, USA) were used at 1/5,000 (vol/vol). The immune complexes were revealed with a chemiluminescent peroxidase substrate (ECL; Amersham, GE HealthCare, Baie d’Urfe, Canada) according to the supplier’s instructions. The DCXR protein signal was quantified by densitometry within the linear range of film exposure and expressed as arbitrary units.

### Immunohistochemistry

Tissue pieces from different epididymal segments were fixed in 4% paraformaldehyde in PBS for 3 days at 4°C and then embedded in paraffin. Six-micrometer histological sections were soaked in 3% hydrogen peroxide in methanol for 20 min to neutralize endogenous peroxidases and then blocked with 5% goat serum in PBS for 1 h. Sections were then incubated overnight (O/N) at 4°C with the rabbit polyclonal antibody against recDCXR at 1/2,000 (vol/vol) in PBS supplemented with 5% goat serum, followed by a 1-h incubation with biotinylated goat anti-rabbit IgG. The immune complexes were revealed with ABC Vectastain kit (Vector Laboratories, Burlingame, CA). Serum from an unchallenged rabbit was used as a negative control. Counterstaining was performed with Harris’s hematoxylin and mounted with Mowiol 4–88 reagent media (Calbiochem, EMB Bioscience, La Jolla, CA)

### Epididymal vesicles (epididymosomes) isolation

Caput and cauda spermatozoa were recovered from epididymal fluid by centrifugation at 1,300 × *g* for 8 min. Supernatants (S1) were retained for epididymosome preparations (Frenette *et al*., 2006). Sperm pellets were resuspendend in 0.15 M NaCl, washed several times by centrifugation and sperm proteins were extracted with 1% SDS in H_2_O. Supernatants (S1) were centrifuged twice at 4,000 × *g* for 20 min at 4°C to eliminate cell debris followed by ultracentrifugation at 120,000 × *g* for 2 h. The resulting pellets were washed once by resuspension in 0.15 M NaCl and ultracentrifuged a second time. The final pellets containing epididymosome fractions were frozen at −80°C until required [21]. These preparations contain small membranous vesicles free of contamination as shown by previous electron microscopy studies [37, 38].

### Proteolytic treatment of epididymosomes, fluid, and recombinant proteins

Epididymosomes, epididymis fluid, or recombinant DCXR proteins were left untreated or were treated with 25 μg/ml trypsin (Sigma) for 40 min at room temperature in 0.15 M NaCl [33].

### Epididymal tissues and spermatozoa sub cellular fractionation

Washed (0.15 M NaCl) spermatozoa (2 × 10^9^) from the caput and cauda epididymides were subjected to nitrogen cavitation at 750 psi for 10 min at 4°C. Cavitated spermatozoa were centrifuged at 10, 000 × *g* for 25 min to pellet the demembranated spermatozoa. The supernatant was recentrifuged at 100, 000 × *g* (32,000 rpm) for 90 min to recover the plasma membrane fraction (pellet) and the cytosolic fraction (supernatant) (adapted from Lawson and collaborators [39]). Demembranated spermatozoa in the 10, 000 × *g* pellet were suspended in HBS, and stored on ice for ultrasonic treatment before western blot analysis.

## Supporting Information

S1 Fig In situ hybridization localization of bovine DCXR mRNA on tissue cryosections of bovine caput (A) corpus (B) and cauda (C) epididymidis. Pictures are at higher magnification (400X). DCXR mRNA staining in blue with the DIG-labeled antisense probe (A, B, C) detected using an anti-DIG antibody coupled to alkaline phosphatase followed by incubation with NBT-BCIP substrate. D panel: corpus section probed with the negative control sense cRNA probes. Counterstaining with neutral red. LU = lumen; EP = epithelium; IT = interstitial tissue V = vessel.
(TIF)

S2 Fig Expression and purification of bovine recombinant DCXR protein. Coomasie blue-stained SDS-PAGE pattern of non-induced cell lysate (lane 1), induced cell lysate (lane 2), and 10 μg of affinity purified DCXR protein (lane 3). M = protein molecular weight marker.
(TIF)

S3 Fig DCXR antibodies characterization. A: Western-blot analysis on 2, 20 and 50 ηg of DCXR rec protein. B: Western blot analysis on 2 and 5 μg of liver; 20 and 40 μg of epididymis protein extract probed with the rabbit anti-bovine recombinant DCXR antiserum, diluted 1 in 10000.
(TIF)

S4 Fig Tissue expression of bovine DCXR protein. Western blot on 25 μg of protein extracted from different bovine tissue. Lane 1: liver; Lane 2: lung; Lane 3: adrenal; Lane 4: brain; Lane 5: spleen; Lane 6: heart; Lane 7: uterus (Days 1–13); and Lane 8: uterus (Days 16–18). Probed with rabbit anti-DCXR antiserum.
(TIF)

S5 Fig Immunohistochemical localization of bovine DCXR protein in Caput (A) corpus (B) and cauda (C) epididymidis using a rabbit anti-DCXR antiserum (A, B and C). DCXR protein is detected as a brown-red staining. (D) caput control with pre-immune rabbit serum. The sections were counterstained in blue with Harris hematoxylin Lu = Lumen; EP = Epithelium; IT = Interstitial tissue; V = Vessel. Arrow indicates staining in an apical cell. Magnification 400X.
(TIF)

S6 Fig Immunohistochemical staining on bovine Caput (A) corpus (B) and cauda (C) epididymidis with a rabbit anti-DCXR antiserum, pictures at higher magnification (1000X). Lu = Lumen; EP = Epithelium; IT = Interstitial tissue; V = Vessel. Arrow indicates staining in an apical cell.
(TIF)

S7 Fig DCXR protein on ejaculated spermatozoa. Western-blot analysis on protein extract from 50 and 100 million of spermatozoa (Spz) from two different bulls (A and B). 20 μg of cauda epididymis protein extract has been used as control. The membrane was probed with the rabbit anti-bovine recombinant DCXR antiserum; dilution 1/10,000 (vol/vol).
(TIF)

S8 Fig DCXR protein and anti-DCXR antiserum sperm—zona pellucida interference assay. In vitro fertilization assay was performed in presence (DCXR-rec) or absence (control) of 20 μg of recombinant DCXR protein (A). Sperm—zona pellucida interference assay was performed in presence of anti-DCXR antiserum (1/500 v/v), the negative control was with control serum. Experiments were in duplicate with 5 oocytes per condition and per trial. No significant difference was observed.
(TIF)
