# Supplementary figures and images for: Dicarbonyl L-Xylulose Reductase (DCXR), a “Moonlighting Protein” in the Bovine Epididymis
Source: PLoS One. 2015 Mar 27;10(3):e0120869. doi: 10.1371/journal.pone.0120869 (PMC4376396; doi:10.1371/journal.pone.0120869)

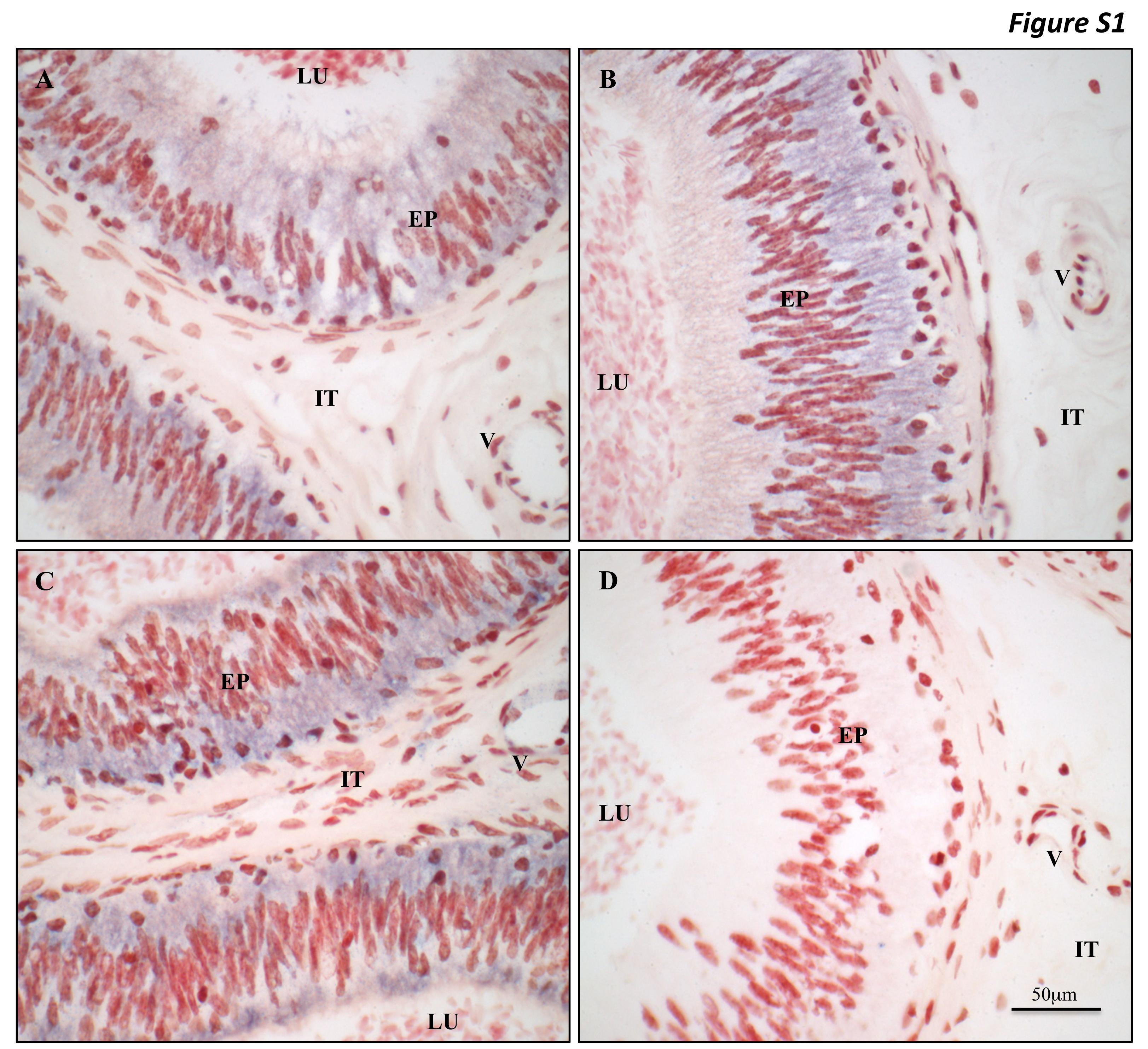

Supplement: S1 Fig — Pictures are at higher magnification (400X). DCXR mRNA staining in blue with the DIG-labeled antisense probe (A, B, C) detected using an anti-DIG antibody coupled to alkaline phosphatase followed by incubation with NBT-BCIP substrate. D panel: corpus section probed with the negative control sense cRNA probes. Counterstaining with neutral red. LU = lumen; EP = epithelium; IT = interstitial tissue V = vessel. (TIF) [file pone.0120869.s001.tif]

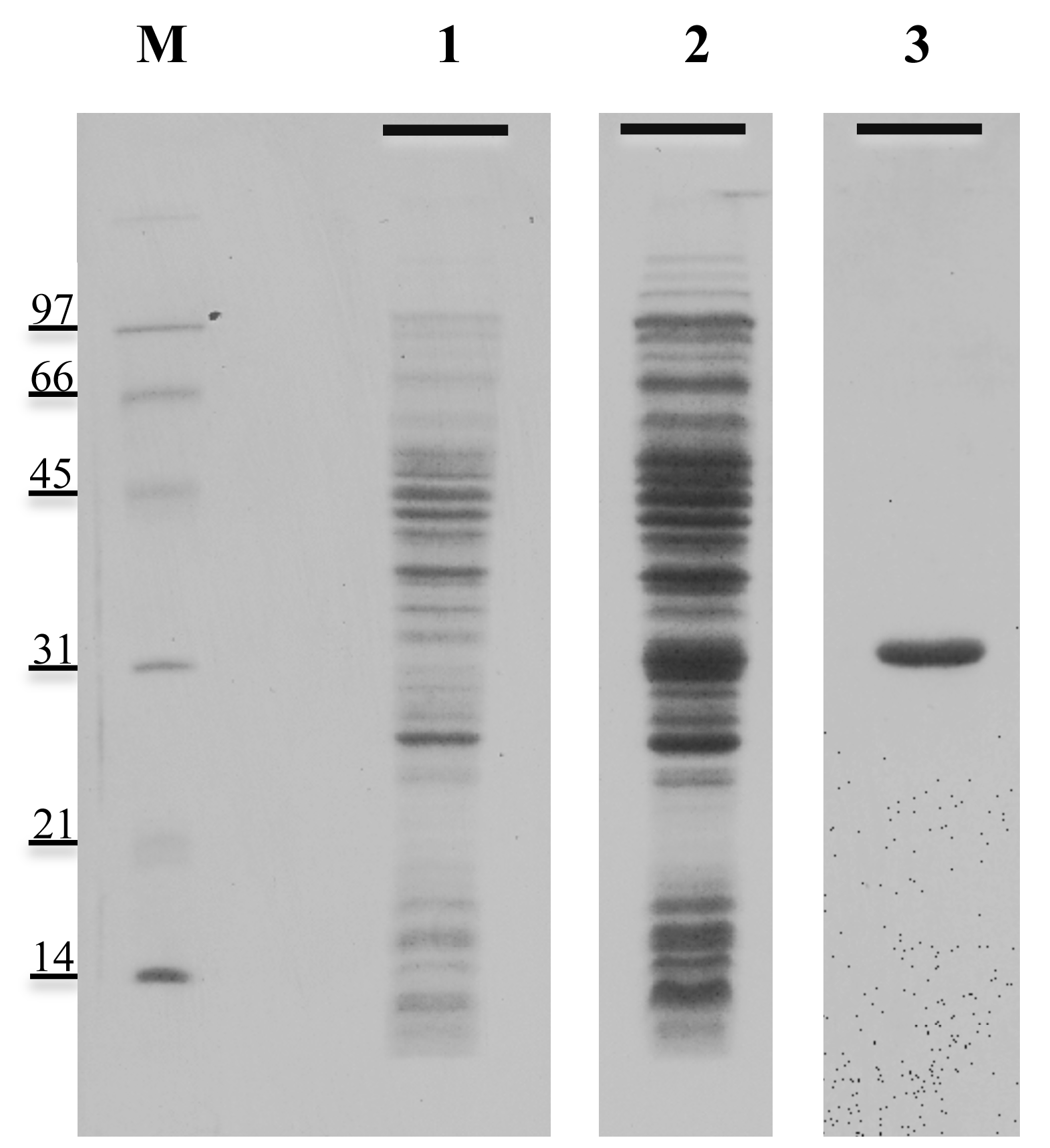

Supplement: S2 Fig — Coomasie blue-stained SDS-PAGE pattern of non-induced cell lysate (lane 1), induced cell lysate (lane 2), and 10 μg of affinity purified DCXR protein (lane 3). M = protein molecular weight marker. (TIF) [file pone.0120869.s002.tif]

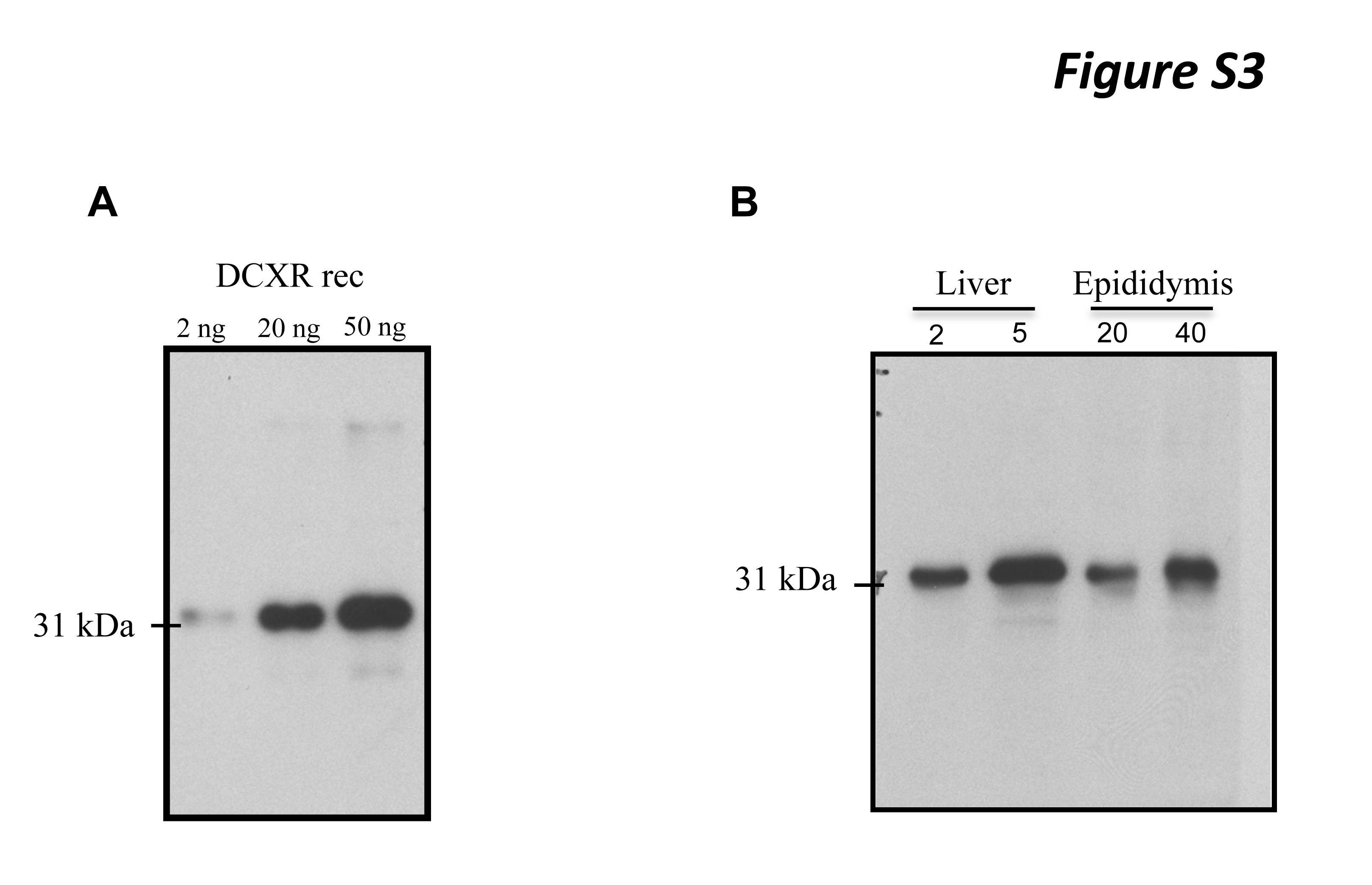

Supplement: S3 Fig — A: Western-blot analysis on 2, 20 and 50 ηg of DCXR rec protein. B: Western blot analysis on 2 and 5 μg of liver; 20 and 40 μg of epididymis protein extract probed with the rabbit anti-bovine recombinant DCXR antiserum, diluted 1 in 10000. (TIF) [file pone.0120869.s003.tif]

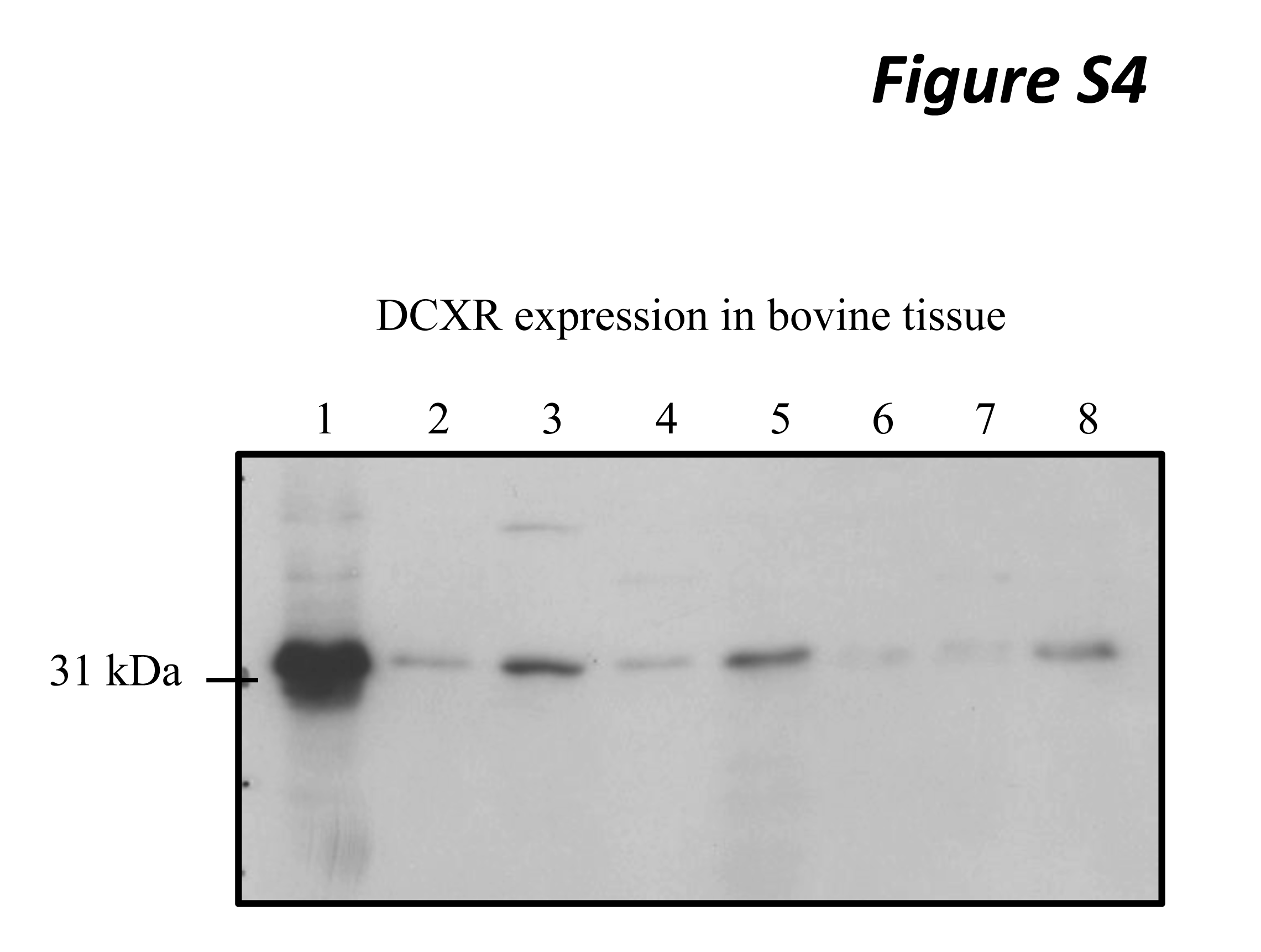

Supplement: S4 Fig — Western blot on 25 μg of protein extracted from different bovine tissue. Lane 1: liver; Lane 2: lung; Lane 3: adrenal; Lane 4: brain; Lane 5: spleen; Lane 6: heart; Lane 7: uterus (Days 1–13); and Lane 8: uterus (Days 16–18). Probed with rabbit anti-DCXR antiserum. (TIF) [file pone.0120869.s004.tif]

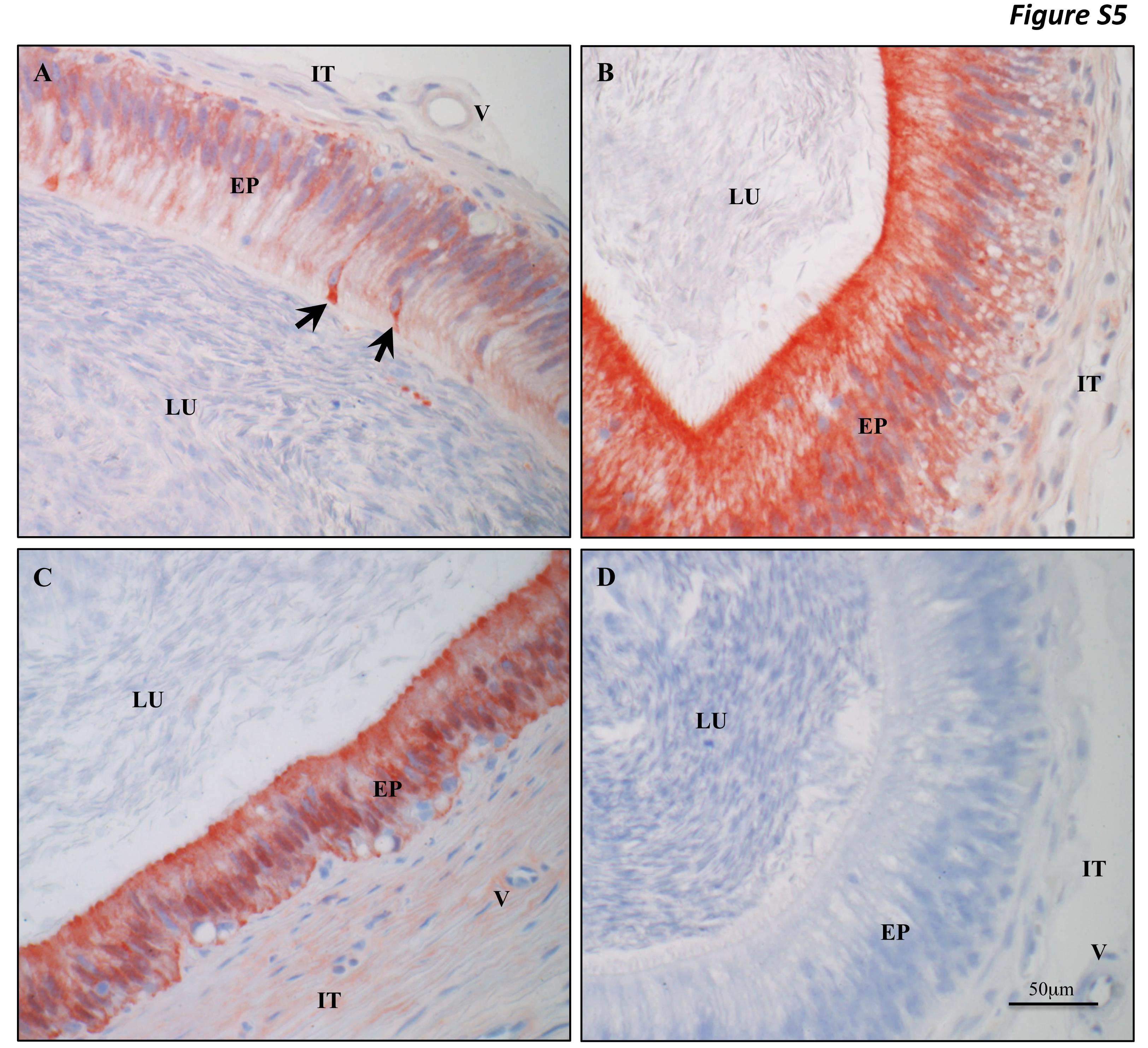

Supplement: S5 Fig — DCXR protein is detected as a brown-red staining. (D) caput control with pre-immune rabbit serum. The sections were counterstained in blue with Harris hematoxylin Lu = Lumen; EP = Epithelium; IT = Interstitial tissue; V = Vessel. Arrow indicates staining in an apical cell. Magnification 400X. (TIF) [file pone.0120869.s005.tif]

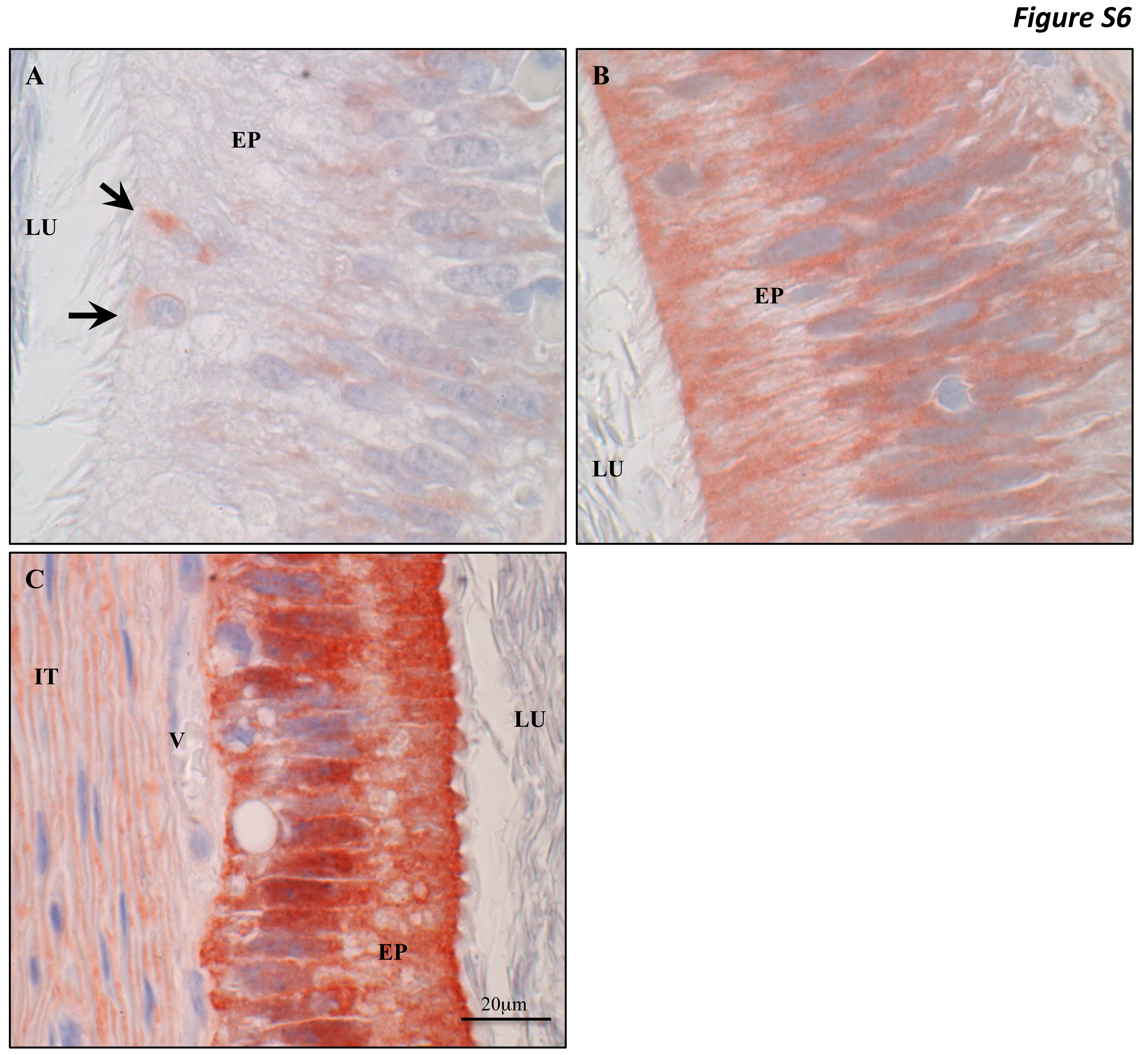

Supplement: S6 Fig — Lu = Lumen; EP = Epithelium; IT = Interstitial tissue; V = Vessel. Arrow indicates staining in an apical cell. (TIF) [file pone.0120869.s006.tif]

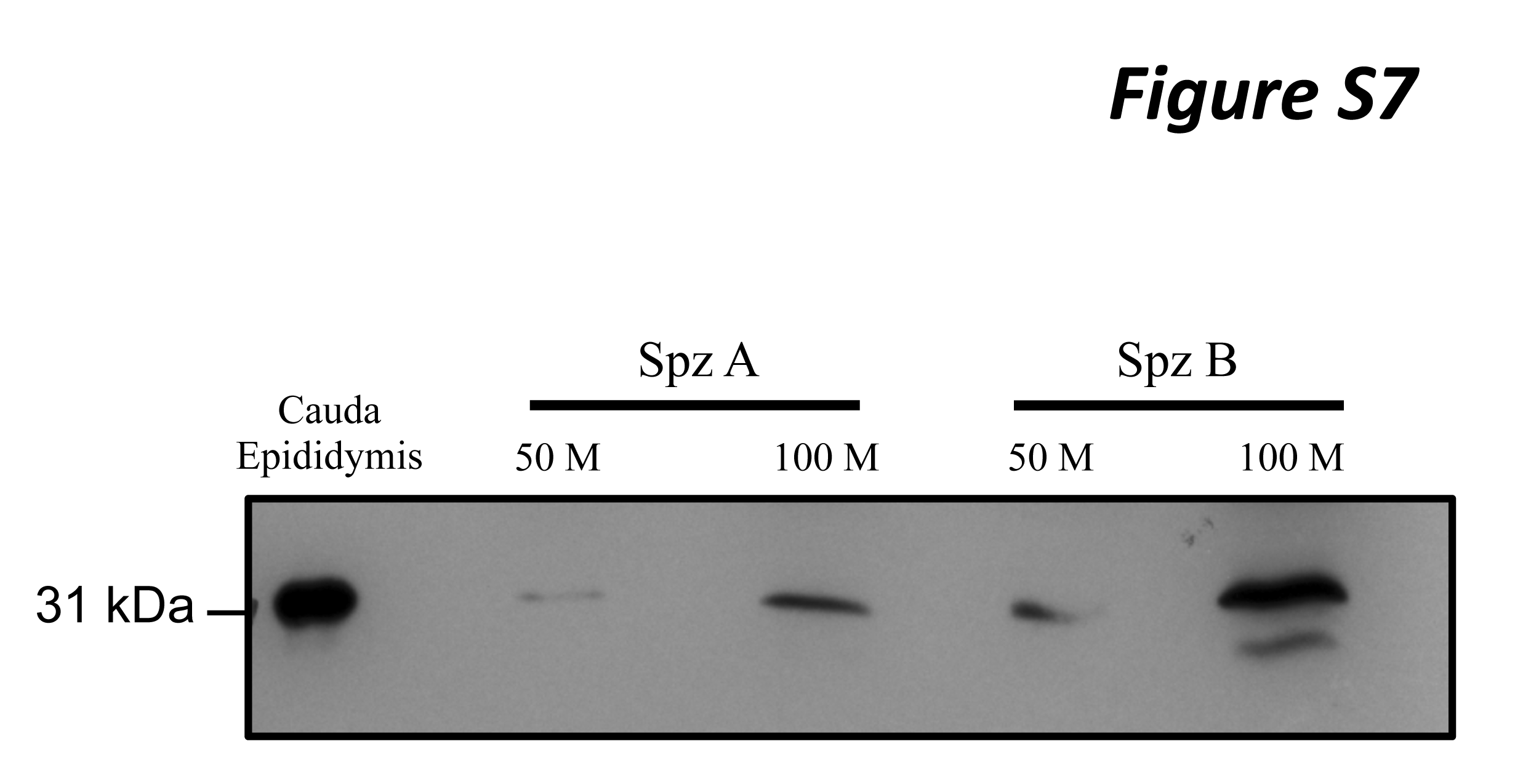

Supplement: S7 Fig — Western-blot analysis on protein extract from 50 and 100 million of spermatozoa (Spz) from two different bulls (A and B). 20 μg of cauda epididymis protein extract has been used as control. The membrane was probed with the rabbit anti-bovine recombinant DCXR antiserum; dilution 1/10,000 (vol/vol). (TIF) [file pone.0120869.s007.tif]

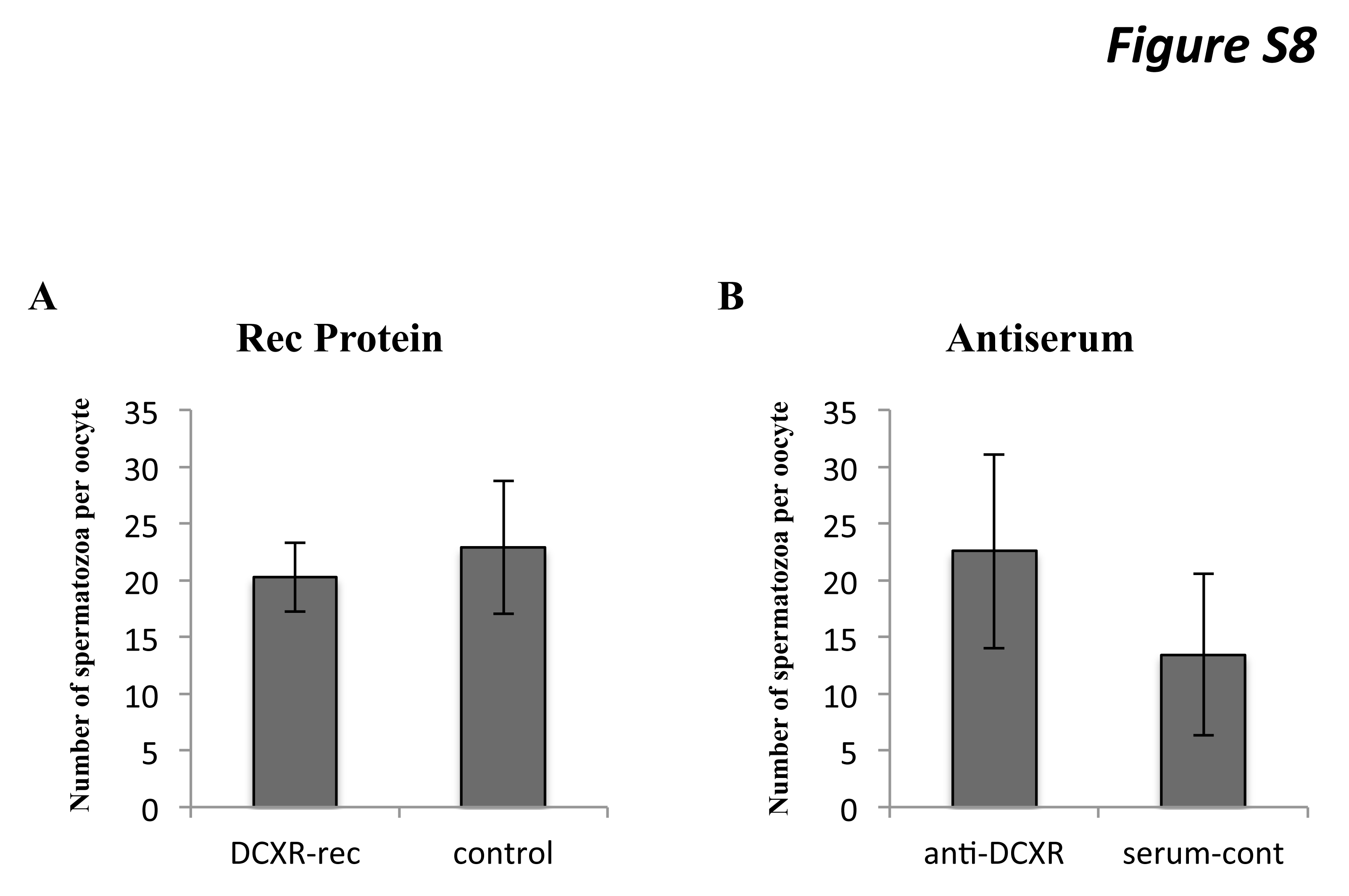

Supplement: S8 Fig — In vitro fertilization assay was performed in presence (DCXR-rec) or absence (control) of 20 μg of recombinant DCXR protein (A). Sperm—zona pellucida interference assay was performed in presence of anti-DCXR antiserum (1/500 v/v), the negative control was with control serum. Experiments were in duplicate with 5 oocytes per condition and per trial. No significant difference was observed. (TIF) [file pone.0120869.s008.tif]
